# Predictors of long-term change of a physical activity promotion programme in primary care

**DOI:** 10.1186/1471-2458-14-108

**Published:** 2014-02-04

**Authors:** Alvaro Sanchez, Gonzalo Grandes, Ricardo Ortega Sánchez-Pinilla, Jesus Torcal, Imanol Montoya

**Affiliations:** 1 Primary Care Research Unit of Bizkaia, Basque Health Service-Osakidetza, Bilbao, Spain; 2 Santa Barbara Primary Care Center, Castilla-La Mancha Health Service, Toledo, Spain; 3 Basauri-Ariz Primary Care Center, Basque Health Service-Osakidetza, Basauri, Spain

**Keywords:** Physical activity, Health promotion intervention, Primary health care, Longitudinal predictors

## Abstract

**Background:**

Further research is needed to improve the evidence regarding determinants of physical activity (PA) as a crucial step to plan higher effective intervention strategies. The goal of the present study is to identify socio-demographic and clinical characteristics of primary care (PHC) insufficiently active patients that are associated with longitudinal changes in the level of physical activity.

**Methods:**

Longitudinal analysis of baseline socio-demographic and clinical predictors of physical activity change in insufficiently active PHC patients who participated in a PA-promoting multi-centre randomized clinical trial conducted from October 2003 through March 2006. The primary outcome measure was the self-reported physical activity assessed with the 7-day Physical Activity Recall (PAR), at baseline, 6, 12 and 24 months. Baseline covariates included sex, age, social class, anthropometric measures and other cardiovascular risk factors or associated diseases (Diabetes, HTA, tobacco use, etc.), and stage of readiness to change PA. Generalized linear mixed models were used to estimate longitudinal association of studied variables on PA change over the three follow-up measurements.

**Results:**

A total of 3691 patients (85% of the 4317 recruited in the trial) with at least one follow-up measurement were included in the longitudinal analysis. At baseline, analysed patients (mean age: 50.6 years; 64.6% women) devoted 34.7 minutes and 2.36 metabolic equivalent hours per week (MET.h/week) to moderate and vigorous physical activity. Older age, male gender, higher social class, lower BMI, diagnosis of diabetes or hypertension, and measurement season were significant predictors of PA longitudinal change. The effect of baseline readiness to change on PA dose was modified by time, showing a positive gradient in favour of those with more readiness to change that increases significantly at 12 and 24 months (p-value interaction < .0001).

**Conclusions:**

Identified baseline characteristics such as readiness to change and risk factors can guide physicians to prioritize time and intervention efforts for maximizing their impact on insufficiently active PHC patients.

## Background

The numerous healthful benefits of regular physical activity participation are well known. Accordingly, it is recommended that adult should perform at least 150 minutes/week of moderate intensity physical activity (PA), 75 minute/week of vigorous-intensity PA or a combination of moderate and vigorous PA [1]. In developed countries, however, a majority of the population does not reach these recommendations and consequently physical activity promotion represents a public health priority [2,3].

Primary health care (PHC) practitioners can play a key role in population health throughout physical activity promotion as they provide on-going care to a large sector of the population [4]. Recent evidence points to a small but positive effect in favour of interventions performed in PHC [5-9]. Yet, the high prevalence of insufficient activity of the population together with the many obstacles family physicians face within a context characterized by work overload and shortage of time and training, supports the need for a targeted strategy for PA promotion in PHC settings [10,11]. As a consequence, selective counselling of patients is currently recommended. Among individual issues to consider for prioritizing might be patient’s risk factors and readiness for change [7,11]. Adapting and targeting the behavioural counselling efforts of family physicians to certain predisposing variables in patients can maximize their impact.

Recent reviews and meta-analysis of PA promotion trials that evaluate the association between patient characteristics and effectiveness of interventions report no clear evidence [12-14]. However, single PA promotion studies have found associations between certain personal and clinical characteristics and greater PA improvement, while mixed evidence for some other variables. For example, regarding gender, although male sex was associated with greater improvement in some studies [15,16], no conclusive evidence has been stated to date [5,12,13]. Something similar occurs with age where some studies have identified age as an inverse correlate [17], but not as a determinant of change in PA promotion interventions [9,14,18]. Regarding people at high risk, single studies show that obesity or higher weight [16,19] are negatively associated with PA change, yet when studies are summarized, their role as a determinant of PA is inconclusive [14,18,20]. An additional constantly remarked criterion for selecting the target population for physician interventions considers the willingness to change [7], as there is convincing evidence of a positive association between baseline PA readiness to change and PA initiation and maintenance from longitudinal studies [14,20].

Further research is needed to improve the evidence regarding determinants of PA as a crucial step to plan more effective intervention strategies, targeted at those who will benefit more. Prospective design studies that assess causal determinants rather than just associations are needed to provide definitive evidence regarding why people are active [14].

The goal of the present study is to identify socio-demographic and clinical characteristics associated with PA longitudinal changes of PHC insufficiently active patients included in a PA promotion intervention trial [21-23]. Trial results showed that general practitioners were effective at increasing the level of PA among their insufficiently active patients during the initial six-months after the intervention, but only in the subgroup of patients receiving repeated prescriptions of PA the effect was maintained in the long-term at 12 and 24 months [22,23]. Once the effect of the intervention has been established, the determination of the extent of which PA changes can be attributed to the presence of certain patient predisposing or modifying variables can guide the adaptation of interventions and intensification of their impact.

## Methods

The present analysis focuses on baseline characteristics as longitudinal prognostic factors of PA increase in insufficiently active PHC patients who participated in a multi-centre randomized clinical trial (from October 2003 to March 2006) conducted in Spain to evaluate the effectiveness of the Experimental Program for PA Promotion (PEPAF) trial [21]. The protocol was approved by the Institutional Clinical Research Ethics Committees (CRECs) for all of the participating centres (ClinicalTrials.gov Identifier: NCT00131079).

### Study participants and procedures

**Figure 1:**
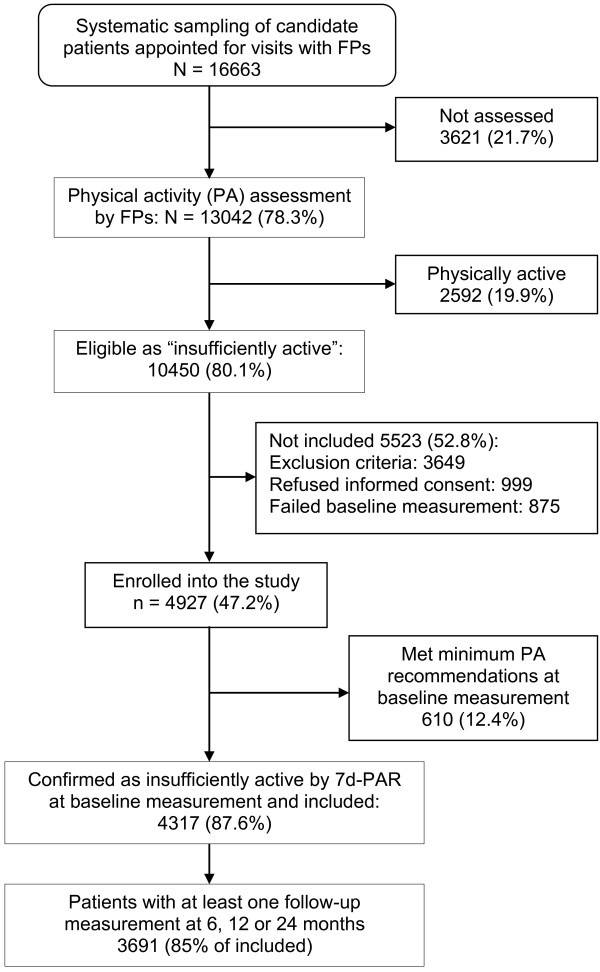
Flow diagram of study participants.

A detailed description of the study setting and participant recruitment is given elsewhere [11,22,23]. In short, a sample of 16663 patients aged 20–80 from those scheduled for an appointment during the recruitment period with the 56 randomized family physicians from 11 PHC centres, was selected by research nurses using systematic sampling. Physicians, after dealing with the reason for the consultation screened the PA level of 13042 selected patients to identify those who did not meet PA recommendations, guided by an interactive web-based algorithm with the following questions: (1) Do you exercise? (2) What type of exercise and how hard do you exercise (providing examples of intensity)? (3) How often and for how long do you exercise? Due to non-attendance, severity of problems, technical difficulties or lack of time, 3621 selected patients were not assessed. Of those assessed, 2592 were identified as active, while 10450 were eligible for the study as they did not meet the recommended aerobic PA levels (moderate-intensity PA for ≥30 minutes 5 day/week or vigorous intensity activity for ≥20 minutes 3 day/week) [1]. The negative answer to the screening questions used by doctors to identify active patients had a predictive value of 87.6% [11]. Further, computer screen shots reminded and guided physicians to review exclusion criteria. After checking existing diagnosis on patient’s Electronic Clinical Record (ECR), 3649 eligible patients were excluded for meeting at least one exclusion criteria, all of them related to contraindications or caution for exercise (cardiovascular or cerebrovascular diseases; neuromusculoskeletal disorders that are exacerbated by exercise; chronic disease with a marked organ or system impairment; uncontrolled metabolic disease; chronic infectious disease; severe emotional distress; complicated pregnancy; and follow-up difficulties); 999 refused to participate when offered an informed consent form; and 875 failed to attend the baseline measurement session. Written informed consent was obtained from all participants involved in the study. Of the 4927 patients who completed the baseline measurement, 610 patients were excluded because they already met the minimum recommended levels of PA, as confirmed in the baseline PA assessment performed by research nurses. Finally, a total of 4317 patients were included in the trial (Figure 1).

The PEPAF intervention has been described in detail [22,23]. In brief, physicians assigned to the PEPAF group provided all patients structured advice on PA benefits and risks of insufficient activity assisted by the web-based software. Additionally, a sub-group of patients that accepted and attended an optional 15 minute consultation set a goal for PA increase at three months with their physician and they agreed to an activity plan, that resulted in a standardized printed prescription and self-monitoring log of the frequency, intensity, duration, and progression of selected activities or exercises. Control group physicians delayed any systematic intervention related to PA until the end of the study. Family Physicians assigned to the intervention group received training on the study protocol, the standardization of the recruitment process and intervention delivery, prescription of physical activity and the theoretical basis of the intervention. In addition to intervention physicians training, quality of counselling was assured by the web-based software which obliged them to advance through several computer screens containing standardized contents of the intervention and registered the process with each patient.

### Measurements and follow-up

The primary outcome measure was the change in PA from baseline to 6, 12 and 24 months after the start of the intervention. This was estimated using the 7-day PA Recall (PAR) semi-structured interview [24] whose validity has been accredited several times over the years, including with Hispanic populations [25]. We also evaluated it in a sample of 160 participants in our study, obtaining good reliability indexes (intra-class correlation 0.6) and a correlation of 0.52 with an objective measurement of activity dose (accelerometer) [26]. The 7-day PAR counts the time spent for all leisure and occupational activities lasting more than 10 accumulated minutes in the 7 days prior to the interview. Minutes per week pertaining to moderate and vigorous PA and the proportion of participants who achieve the minimum recommended PA levels are directly calculated, while weekly activity dose in metabolic equivalents hour per week (MET*h/wk) is estimated by multiplying the hours devoted to activities of moderate, hard, and very hard intensity by the corresponding METs, i.e. 4, 6, and 10, respectively. The PAR was the reference standard used to independently confirm if patients identified as insufficiently active by the physician did or did not meet the minimum public health PA recommendations.

#### Covariates

The assessment of PA stage of change that represents ordered categories of motivational readiness to change was based on the recommendations of Reed et al. [27]. Using a self-administered questionnaire that included the abovementioned definition of regular PA, the participants had to select the statement that best described his or her current status, from among 5 choices: “No, I do not exercise regularly and I do not intend to do it in the next 6 months” (Precontemplation); “I do not exercise regularly, but I intend to do it in the next 6 months” (Contemplation); “I do not exercise regularly, but I intend to do it in the next 30 days” (Preparation); “I have been exercising regularly for less than 6 months” (Action); “I have been exercising regularly for more than 6 months” (Maintenance).

Height was measured with a wall stadiometer and weight was measured with a calibrated digital scale. Body mass index (BMI) was calculated as kilograms per meter squared. Cardiovascular risk factors (diabetes, high blood pressure, dyslipidemia) were reported by the family doctors after reviewing patients’ records. Tobacco use was obtained by self-report and alcohol consumption was identified using the Spanish version of the AUDIT, which defines an at-risk drinker as one who scores 8 or more points [28]. Social class and level of education were recorded and classified following the recommendations of the Spanish Society of Epidemiology [29].

Trained nurses working in exercise laboratories who performed baseline and follow-up measurements at 6, 12 and 24 months were blinded to the allocation group of the participants. The quality of measurements was assured through the training of research nurses, a pilot study and double data entry into a central OracleTM database. A telephone recall system was used to improve follow-up rates of patient measurements. Quality control was performed by the coordinating centre (PHC Research Unit of Bizkaia) with daily online supervision and feedback to nurses regarding the study process and data entry, monthly progress reports, and regular meetings every four months with the collaborating investigators and research nurses.

### Analysis

Generalized linear mixed models were used to estimate longitudinal association of the variables studied on PA change over the three follow-up measurements, to take into account the repeated measurements for each patient and also the hierarchical structure of data, with patients nested in doctors and health centres (SAS PROC MIXED and GLIMMIX ver. 9.2, SAS Institute, Cary, NC, USA, 2009; SAS code available upon request). These models were linear for changes in PA dose (MET*h/wk), and logistic for achievement of the minimum recommended PA levels. Time evolution was considered in these models as a categorical variable (follow-up point), with several correlated measurements for each individual. This option was chosen as it is the less restrictive and provided a better fit to our data.

As the present study tries to identify and to assess predictors of longitudinal PA increase regardless of the intervention received, the intervention group, the time of measurement and intervention-by-time interaction were included as fixed effects in the models. Patients, physicians and centres were included as random effects in the intercept and in the slope of the different repeated measurements. PA determinants included baseline values of the outcome variables, socio-demographics, risk factors, baseline stage of change and the season of the year in which the measurements were made. Different covariance structures were used for the repeated observations on the same patient, doctor and centre and restricted maximum likelihood ratio tests were used to determine the best covariance structure for our data. Likewise, to simplify the fixed effects structure, likelihood ratio tests were used following backward, forward and stepwise strategies (significance criterion p < 0.05). No imputation method was used to handle the missing data since longitudinal mixed models based on maximum likelihood estimation used in this paper are more appropriate to deal with missing data than common imputation methods such as last observation carried forward, complete case analysis or other possible forms of imputation [30].

## Results

A total of 3691 patients with at least one follow-up measurement (85% of the 4317 recruited in the study) were included in the longitudinal analysis. The mean age was 50.6, 64.6% were women, the average time devoted to moderate or vigorous activities was 34.7 min/wk and the mean weekly activity level was 2.4 MET*h/wk. A third of the sample had low levels of education and more than half were catalogued in the “manual worker” social class. Regarding risk factor variables, 25% of the sample was obese, 30% were current smokers and 8% were diagnosed with diabetes. Only a quarter of the sample was considering to change PA levels in the next month and 6% and 9% reported to be in the ”action” or “maintenance” stage of change, respectively (see Table 1). Baseline, 12 and 24-month measurements were mostly made in autumn and winter, whereas the majority of 6-month measurements were performed in spring and summer. Overall, 21.7%, 24.1% and 28.0% of baseline insufficiently active patients met PA recommended levels, at 6, 12 and 24-month respectively (results not shown).

**Table 1:** Socio-demographic characteristics of insufficiently active primary care patients included in the PEPAF trial

	**Included patients N =3691**
**Physical activity**	
Physical activity dose, mean METs*h/wk (sd)	2.4 (6.0)
Moderate and vigorous activity, mean minutes/week (sd)	34.7 (87.0)
**Cardio-respiratory fitness**	
VO2max, mean ml/kg/minute (sd)	24.5 (8.2)
***Socio-demographic variables***	
Mean age (sd)	50.6 (14.7)
Female	2384 (64.6)
Educational level	
None	219 (5.9)
Elementary school	1138 (30.8)
Middle or high school	1706 (46.2)
University studies	628 (17.1)
Social class^a^	
Manager large enterprise	256 (6.9)
Manager small enterprise	403 (10.9)
Intermediate employee	1092 (29.6)
Manual worker	1940 (52.6)
***Risk factors***	
Diabetes	308 (8.3)
Hypertension	939 (25.4)
Dyslipidemia	810 (21.9)
Body mass index (BMI)	
Normal (< 25 kg/m^2^)	734 (32.6)
Overweight	933 (41.5)
Obese (≥ 30 kg/m^2^)	581 (25.8)
Smoking	
Current smoker	1081 (29.3)
Former smoker	702 (19.0)
No smoker	1908 (51.7)
At-risk drinker	187 (5.1)
Physical activity stage of change	
Pre-contemplation	1036 (28.1)
Contemplation	1240 (33.6)
Preparation	936 (25.3)
Action	147 (4.0)
Maintenance	332 (9.0)

^a^Social class categorization based on occupation and work position as recommended by the Spanish Society of Epidemiology [29]: Class IV to V includes non-qualified and qualified manual workers; Class III includes the administrative workforce, supervisors and free-lance workers; Class II includes managers of company with less than ten employees, professionals with first level university degree, senior technicians, artists and sportsmen/women; Class I includes managers of public organizations or private companies with more than ten employees, professionals with second and third level university degrees.

Values are frequencies (%), unless otherwise indicated.

Table 2 shows the association of demographic and clinical variables and the longitudinal change in activity dose measured at base-line. Age, sex, social class, BMI, diabetes, baseline PA level, measurement season and stage of change remained as significant predictors of PA change over 24 months. Being older than 50 years old, male gender and pertaining to the lowest social class was positively associated with a greater increase in activity dose. Additionally, those diagnosed with diabetes, and overweight and normal weight patients as compared to obese patients, also showed a higher change in activity dose. The activity level was higher in warmer seasons compared to winter. However the abovementioned associations were not modified by time and remain constant in each of the follow-up endpoints.

**Table 2:** **Baseline level predictors of longitudinal change**^**a **^in activity dose (MET*h/wk) among insufficiently active primary care patients included in the PEPAF trial

	Adjusted MET*h/wk change (95% CI)	**P-value**
***Time independent predictors***		
**Age**		0.005
< 50	Reference	
≥ 50	1.13 (0.34 to 1.92)	
**Sex**		<.0001
Female	Reference	
Male	3.08 (2.31 to 3.85)	
**Social class**		0.004
Manual worker	Reference	
Manager large enterprise	2.27 (0.74 to 3.79)	
Manager small enterprise	1.07 (−0.48 to 2.62)	
Intermediate employee	1.39 (−0.36 to 3.15)	
**BMI**		0.0001
Obese	Reference	
Normal weight	1.76 (0.78 to 2.75)	
Overweight	1.77 (0.90 to 2.64)	
**Diabetes**	1.92 (0.57 to 3.24)	0.0051
**Measurement season**		<.0001
Winter	Reference	
Spring	3.29 (1.65 to 4.94)	
Summer	1.64 (0.06 to 3.21)	
Autumn	1.77 (0.93 to 2.60)	
***Time dependent predictors***		
**Baseline readiness to change**		<.0001
Pre-contemplation	Reference	
Contemplation		
6 month	1.40 (0.20 to 2.59)	
12 month	1.04 (−0.35 to 2.44)	
24 month	1.27 (−0.31 to 2.88)	
Preparation		
6 month	1.40 (0.11 to 2.71)	
12 month	0.58 (−0.95 to 2.12)	
24 month	1.72 (−0.04 to 3.49)	
Action		
6 month	1.41 (−1.10 to 3.91)	
12 month	4.03 (1.04 to 7.02)	
24 month	2.40 (−0.89 to 5.70)	
Maintenance		
6 month	3.62 (1.79 to 5.45)	
12 month	6.47 (4.33 to 8.61)	
24 month	7.95 (5.54 to 10.37)	

^**a**^Adjusted for group by time interaction, reported elsewhere [23].

**Figure 2:**
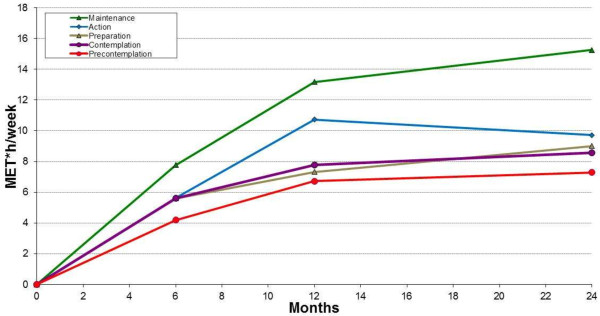
Longitudinal change in activity dose (MET*h/week) by baseline stage of change. Figure legend: Adjusted for group by time interaction, reported elsewhere [23].

By contrast, the effect of baseline readiness to change on activity dose is modified by time. Figure 2 shows the longitudinal effect of baseline readiness to change on activity dose adjusted change and illustrates the modification of the estimated increases of patients catalogued in the different stages in each follow-up endpoint. Overall, change in activity dose increases over time for every stage of change. Additionally, there is a positive gradient in the effect of baseline readiness to change and PA change that increases significantly at 6, 12 and 24 months (p-value interaction < .0001). That is, as time passes, the higher the readiness to change the higher the increase in PA level. At the 12 month follow-up, those patients that reported to be in maintenance at baseline, significantly differed from precontemplators (Adj. change?=?6.45 Met*h/week, 95% CI 4.31 to 8.59). At the 24 month follow-up point, those in maintenance presented the highest increase and significantly differed by 7.98 Met*h/week (5.56 to 10.39) from those in precontemplation.

Table 3 describes baseline prognostic factors of meeting PA recommendations. None of the variables interacted with time thus showing a constant effect that does not change all along the follow-up endpoints. Specifically, those patients aged 50 years or more had a 22% higher probability of meeting guidelines in any follow-up point compared to younger patients. Male gender is also associated with greater change (AOR?=?1.21, 95% CI 1.07 to 1.37). Regarding clinical risk factors, overweight (AOR?=?1.27, 1.16 to 1.37) and normal weight patients (AOR?=?1.20, 1.01 to 1.42) as compared with obese patients, and those diagnosed with diabetes (AOR?=?1.36, 1.22 to 1.48) or hypertension (AOR?=?1.18, 1.05 to 1.29) showed a higher probability of meeting PA recommendations. Measurement season is also associated with the probability of meeting guidelines (p < .0001), with those measured in spring and summer showing a greater effect. Finally, patients in the preparation, action or maintenance baseline stage of change had a higher probability of meeting guidelines in any follow-up time that those who reported to be in the “precontemplation” stage of change. Analyses restricted to those 2857 patients that completed the repeated follow-up yielded similar results regarding which variables remained as significant predictors of and the magnitude and direction of their associations with activity dose change (Met*h/week) and proportion meeting PA recommendations.

**Table 3:** **Baseline level predictors of longitudinal change**^**a **^**in the proportion meeting physical activity recommendations among insufficiently active primary care patients included in the PEPAF trial**

	**Meet PA level**	
**Variable**	**Adjusted OR (95% CI)**	**p-value**
**Age**		0.0002
< 50	1.00	
≥ 50	1.22 (1.11 to 1.32)	
**Sex**		0.002
Female	1.00	
Male	1.21 (1.07 to 1.37)	
**BMI**		0.0004
Obesity	1.00	
Underweight	1.54 (0.80 to 2.96)	
Normal weight	1.20 (1.01 to 1.42)	
Overweight	1.27 (1.16 to 1.37)	
**Diabetes**	1.36 (1.22 to 1.48)	<.0001
**Hypertension**	1.18 (1.05 to 1.29)	0.001
**Baseline readiness to change**		
Precontemplation	1.00	<.0001
Contemplation	1.10 (0.94 to 1.29)	
Preparation	1.22 (1.03 to 1.45)	
Action	1.70 (1.26 to 2.30)	
Maintenance	1.92 (1.56 to 2.48)	
**Measurement season**		<.0001
Winter	1.00	
Spring	1.86 (1.40 to 2.47)	
Summer	1.39 (1.06 to 1.82)	
Autumn	1.18 (1.04 to 1.33)	

^**a**^Adjusted for group by time interaction, reported elsewhere [23].

## Discussion and conclusions

Findings of this study point to several socio-demographic and clinical characteristics associated with greater improvement in PA change of insufficiently active PHC patients included in a PA promotion trial conducted in PHC. Specifically, older age, male gender, higher social class, lower BMI, diagnosis of diabetes or hypertension, and baseline readiness to change are positively associated with longitudinal changes in PA dose or/and with meeting PA recommended levels. Recent studies have reported decreases in cardiovascular risk associated with as little as 45 to 60 minutes of walking per week or 2.6 to 3.9 MET-h/wk [1], figures similar to some effects observed on this study (e.g., change in males vs. females). PA interventions in PHC settings are an effective way to improve PA of insufficiently active patients [7,8]. However, the circumstances that currently prevail in most healthcare systems impede PHC physicians to implement PA interventions to all of their insufficiently active patients and support the need for a targeted strategy for PA promotion in PHC settings [7,11]. The evidence provided by the present study regarding longitudinal determinants of PA can be useful for general practitioners in regard to prioritization of time, effort, and targeting intervention strategies to specific population subgroups who will most likely change their insufficiently active behaviour.

As reported in previous studies on PA determinants [15,16], men in our study achieved greater change in activity dose and were more likely to meet PA recommendations than women. Yet, in contrast to conclusions of other studies or systematic reviews in which no effect or positive effect for younger patients have been stated [9,14,18], patients aged ≥50 years showed greater improvements compared to younger patients in our study. A possible explanation of this association could be that older people who have greater free time at their disposal due to less work occupation or demanding obligations may face fewer obstacles to meet health behaviour recommendations [31]. In addition, results confirm that a higher social class is associated with higher changes in activity dose [16]. Lastly, another factor associated with activity change in our study is the season where the follow-up measurement has been carried out. Specifically, higher changes are observed in warmer months, especially in spring as compared to winter. This result is not surprising as it has been previously reported [32,33].

Regarding clinical variables, and consistent with what has been previously documented, normal weight and overweight patients showed greater improvements and were more likely to achieve recommended levels compared to obese patients [16,19]. We also found that those diagnosed with diabetes had greater increases compared to non-diabetic patients. This association is somewhat consistent with previously documented associations found when comparing pre-diabetic and diabetic patients [34] or when comparing the PA level of patients with previously undiagnosed (screen-detected) and those with previously diagnosed (known) type 2 diabetes [35]. In addition, patients diagnosed with high blood pressure were also more likely to meet recommendations. Jointly, it seems that patients with chronic diseases might be more receptive to PA counselling than “healthy” patients or the general population [13,36].

Literature on the evaluation of the validity of the stage of change construct as a predictor of PA level is scarce, yet recent evidence has identified it as a determinant of PA [14,20]. As has been already shown in other studies [37,38], the results of our study reveal a positive effect of having a more advanced baseline readiness to change on PA improvement at 12 and 24 months. Moreover, this positive effect of baseline stage of change seems to be modified by the passing of time and maximized in those with more willingness to change. As stated in the analysis section, statistical models were controlled for intervention group assignment [23], pointing out that the results regarding baseline readiness to change are independent of the intervention received. Thus, baseline readiness to change by itself seems to be a prognostic factor of PA change.

Some strengths and limitations of the present study should be commented on to clearly interpret results. Although 17% of eligible patients refused to participate or did not attend to baseline measurements, the study has recruited a large sample of insufficiently active patients not especially motivated to change (near to 60% of included patients). The systematic sampling used for selecting eligible patients in order to minimise a potential recruitment bias in the study, together with their detection by 56 GPs under routine practice conditions in 11 cities from 8 different autonomous regions in Spain, give the results a great capacity for generalization to insufficiently active patients seen in primary care. Patient characteristics are representative of the common socio-demographic and clinical characteristics seen in PHC [11]. The main limitation of the study is the self-reported measurement of PA, which may be associated with recall and social desirability biases. Although it would have been more valid to use objective measures of PA, this would have been impractical in such a large-scale study with thousands of patients and multiple measurements. However, the 7-day PAR has been shown to correlate well with objective measures in previous studies and in a sub-sample of our study population [26]. Also, although our study included some important socio-demographic and clinical variables, numerous other variables were not measured and may be important determinants of PA, including environmental, social and other psychological factors [39]. Trial recruitment procedures and PA measurement may have affected associations reported in the present study. However, recruitment and measurement processes are also present in observational studies, and these processes have been linked to changes in behaviour [40].

Our findings have identified baseline patient personal and clinical characteristics that could help PHC professionals ascertain which patients are most likely to change PA level. Specifically some clinical risk factors (e.g., blood pressure, BMI, etc.) and readiness for change be useful indicators to tailor intervention programs and direct efforts and resources more efficiently and effectively [7,11]. But at the same time, findings point out those patients in whom future interventions should improve, such as women, those with lower social class or obese patients, among others. PA promotion programs within PHC can enable insufficiently active patients to increase their PA levels, yet further investigation is warranted on the best approaches to finally integrate such complex interventions in routine care and make them sustainable [10,23].

## Competing interests

The authors declare that they have no competing interests.

## Authors’ contributions

GG conceived the idea and is the study guarantor. He is primarily responsible for the study design and planning, obtained funding, and was responsible for project coordination and supervision, analysis and interpretation of results and manuscript preparation. AS, RO, JT, and IM collaborated in the study design, obtained funding, and were responsible for study coordination, interpretation of results and manuscript preparation. GG, AS, RO, JT and IM were responsible for the analysis of results and critically reviewed the manuscript. All contributors approved this version submitted for publication to *BMC Public Health.*

## Pre-publication history

The pre-publication history for this paper can be accessed here:

http://www.biomedcentral.com/1471-2458/14/108/prepub

## PEPAF group members

Director Team: Primary Care Research Unit of Bizkaia, Basque Health Service-Osakidetza (principal investigator: Gonzalo Grandes; co-investigators: Jesus Torcal, Ricardo Ortega Sanchez - Pinilla, Alvaro Sanchez, Kepa Lizarraga, Javier Serra, Imanol Montoya). Basque Health Service-Osakidetza: Basauri-Arız Health Center (Angel Fernandez, Victor Manuel Lopez, Lourdes Marijuan, Begoña Etxeguren, Victor Landa, Jesus Martınez, Ma Mercedes Dıez, Juan Ramon Lejarza, Judith Gonzalez), Galdakao Health Center (Vidal Salcedo, Idoia Ibañez, Agurtzane Ortego, Pedro Iraguen, Pilar Echevarria, Virginia Villaverde), Algorta Health Center (Amaia Ecenarro, Ma Teresa Uribe, Carmen Moral, Eguskiñe Iturregui, Ana Belen Fernandez). Andalusian Health Service: Camas Health Center - Seville (Jose Marıa Paez, M Angeles Tarilonte, Concepcion Molina, Vicente Rodrıguez, Isabel Villafuente, Mercedes Alvarez). Balearic Islands Health Service: Dalt Sant Joan Health Center-Mahon (Andreu Estela, Jose Marıa Coll, Angels Llach, Josep Ma Masuet, Ana Moll, Monica Pons). Catalan Health Service: Serraparera Health Center - Barcelona (Bonaventura Bolibar, Agusti Guiu, Amadeu Dıaz, Xavier Martınez, Ma Dolores Hernandez, Jose Ignacio Olivares, Francisco Hernansanz, Rita Ayala, Ana Cascos). Castilla - la Mancha Health Service: San Fernando Cuenca III Health Center – Cuenca (Vicente Martınez, Ma del Carmen Garcıa, M Angeles Gabriel, M Luscinda Velazquez, Natividad Ortega, M Jesus Segura, Rodrigo Cerrillo, Patricia Lopez). Castilla y Leon Health Service: Casa Barco Health Center – Valladolid (Carmen Fernandez, Amparo Gomez, Miguel Angel Dıez, Ruperto Sanz, Luis Miguel Quintero, Jose Ignacio Recio), La Alamedilla Health Center – Salamanca (Luis Garcıa, Jose Antonio Iglesias, Manuel Gomez, Emilio Ramos, Pilar Moreno, Yolanda Castaño, Nadia Carrillo). Galician Health Service: Sardoma Health Center – Vigo (Pilar Ganoso, Luciano Casariego, Manuel Domınguez, Jose Ramon Moliner, Fernando Lago, M Concepcion Cruces, Marisa Enrıquez). Madrid Health Service: Guayaba Health Center – Madrid (Tomas Gomez Gascon, Javier Martınez, Jose Antonio Granados, M Angeles Ferna’ndez, M Isabel Gutierrez, Carlos San Andres, Concepcion Vargas-Machuca, Cristina Dıaz).
